# The Effect of Health Literacy Interventions on Self-management in Chronic Diseases: A Systematic Review

**DOI:** 10.1093/abm/kaaf073

**Published:** 2025-10-31

**Authors:** María-Francisca Cabezas, Gabriela Nazar, Adelita V Ranchor, Coby Annema

**Affiliations:** University of Groningen, University Medical Center Groningen, Department of Health Sciences, Health Psychology Section, PO Box 30.001 9700 RB Groningen, The Netherlands; Universidad de Concepción, Department of Psychology, Faculty of Social Sciences, Concepción, 4030000, Chile; University of Groningen, University Medical Center Groningen, Department of Health Sciences, Health Psychology Section, PO Box 30.001 9700 RB Groningen, The Netherlands; University of Groningen, University Medical Center Groningen, Department of Health Sciences, Nursing Science, PO Box 30.001 9700 RB Groningen, The Netherlands

**Keywords:** health literacy, self-management, intervention, chronic diseases, systematic review

## Abstract

**Background:**

Patients need cognitive and social skills in order to be able to make informed healthcare decisions. Health literacy (HL) encompasses these skills, enabling individuals to manage their conditions and adapt to challenges. HL interventions in patients could be a powerful way of optimizing self-management (SM) in individuals with chronic diseases.

**Purpose:**

To examine the efficacy of HL interventions on the medical, emotional, and role management domains of SM, theoretical foundations, conceptualizations of HL and SM, and intervention components.

**Methods:**

Health literacy interventions aimed at increasing SM in adults (≥18 years) with chronic diseases were considered. A database search was conducted in PubMed, Scopus, SciELO (Scientific Electronic Library Online), and Web of Science from 2014 to 2024. Results were reported according to the Preferred Reporting Items for Systematic Reviews and Meta-analyses. Risk of bias was assessed using the Joanna Briggs Institute Checklist.

**Results:**

Fourteen studies were included. Interventions improved 3 components of medical management: medication adherence, disease knowledge, and self-efficacy. However, the effects on adherence to diet and exercise regimens, also part of medical management, were inconclusive. Emotional and role management received limited attention. There was inconsistency between HL definitions and instruments. Most interventions were delivered through in-person sessions. Overall, studies showed moderate risk of bias, which may have influenced the results.

**Conclusions:**

Theory-based interventions, methodological consistency, and comprehensive HL and SM measures are needed to understand interventions’ effectiveness. To support behavioral change, HL interventions must address emotional and role management. Future high-quality research is required to determine optimal strategies for strengthening SM through HL interventions.

## Introduction

Non-communicable diseases (NCDs) are the most important determinant of disability and mortality worldwide. In 2021, approximately 43 million deaths were attributed to NCDs, equivalent to 75% of non-pandemic-related deaths globally.^1^ As the number of chronic conditions in an individual increases, risks of mortality, functional deterioration, and unnecessary hospitalizations also rise.^2^^,^^3^ Moreover, adults with multiple chronic diseases experience poorer health-related quality of life and higher healthcare costs compared to those without chronic diseases.^4–6^ This growing burden has driven a shift in chronic disease management, where patients have transitioned from passive recipients of care to active participants in managing their health in collaboration with healthcare providers.^7^^,^^8^

Self-management (SM) can be defined as the ability of an individual, together with family, community, and healthcare professionals, to manage symptoms, treatments, lifestyle changes, and psychosocial, cultural, and spiritual consequences of health conditions to maintain a satisfactory quality of life.^9^ Lorig and Holman’s framework^10^ described SM tasks as falling into 3 categories, including medical management, which incorporates such tasks as effectively using medication, following dietary or exercise recommendations, and monitoring medical aspects of one’s health; role management, encompassing the adjustment to shifts in domestic, social, and vocational domains; and emotional management, including coping with the emotional changes associated with living a chronic disease. In this model, 6 core SM skills facilitate the accomplishment of SM tasks, including problem-solving, decision-making, taking action, using resources, communication with health personnel, and self-tailoring.^10^

Extensive research supports the positive impact of SM on health outcomes across different chronic diseases. For instance, Kumah et al.^11^ found that combining primary care and SM significantly improved glycemic control. In addition, effective SM was found to reduce HbA1c (glycated hemoglobin) levels in type 2 diabetes mellitus,^12^^,^^13^ aid in weight reduction for persons with obesity,^14^ decrease exacerbations in chronic obstructive pulmonary disease patients,^15^ and lower hospitalization rates in heart failure patients.^16^ These findings have led to the development of interventions aimed at strengthening SM,^17–21^ which are generally effective in improving patients’ health outcomes,^22^^,^^23^ and combine health education, goal setting, and problem-solving training to achieve these outcomes.^10^^,^^24^

A key factor that might be associated with reducing barriers to effective disease management is health literacy (HL), which is defined as the ability to obtain, process, and understand basic health information and services.^25^ According to Nutbeam,^26^ HL can be divided into 3 levels: functional, communicative, and critical HL. All 3 levels together comprise complex skills that enable an individual to extract, evaluate, and apply health-related information. Inadequate HL limits the ability to access and use health information and act on public health alerts and is associated with worse health outcomes and higher healthcare costs.^22^^,^^27^ Communicative and critical HL are essential for SM in patients, especially in disadvantaged groups with low HL, by empowering active and informed participation in healthcare.^28–32^

Health literacy interventions aim to improve the use of health information and address topics such as health behaviors, treatment adherence, and SM skills.^31^ Key elements contributing to their effectiveness include cultural appropriateness, individual tailoring, and active discussion.^32^ HL interventions are delivered through various formats, including counseling, tele-­education, group-based learning, and conversational approaches.^33^^,^^34^ However, the diversity of these interventions makes it difficult to determine the most effective components for disease management.

While several observational studies have linked low HL levels to poor SM, evidence from interventional studies remains unclear. Some studies suggest that HL interventions among patients improve individuals’ ability to manage the medical, emotional, and role domains of SM.^23^^,^^30^ However, other studies report no effect or negative outcomes.^35^^,^^36^ A recent systematic review identified several factors contributing to these inconclusive results, including inconsistencies in the definitions and measurements of HL, variations in theoretical frameworks, and differences in the intervention components.^34^ Conceptual models, such as Paasche-Orlow and Wolf,^37^ Sørensen et al.,^25^ and Wolf et al.^38^ explain causal pathways between HL and outcomes, by identifying mediating factors such as SM, patient-provider communication, and access of health care. These models help to understand the process by which HL affects health. However, as the field evolves, multiple definitions and frameworks for HL and SM have emerged, contributing to conceptual ambiguity and a lack of consensus.^26^^,^^37^^,^^38^ Advancing the field requires developing standardized, theory-based frameworks with unified definitions and measures.

To address this gap, this study aimed to synthesize evidence on the effectiveness of HL interventions on SM across various chronic conditions. The first aim was to examine the efficacy of HL interventions in medical, emotional, and role management domains of SM. The second aim was to examine the theoretical foundations and conceptualization of HL and SM (variable definitions and instruments) and key components of HL interventions.

## Methods

### Approach and Review Registration

This systematic review was conducted following the Preferred Reporting Items for Systematic Reviews and Meta-Analysis (PRISMA) guidelines^39^ and the Cochrane Handbook for Systematic Reviews of Interventions.^40^ A quality assessment using the Joanna Briggs Institute (JBI) critical appraisal tools was carried out to safeguard the standards of the studies, including objectivity, systematization and replicability of the results. PRISMA checklist is provided in Supplementary Material 1. This systematic review has been registered in PROSPERO on April 26, 2024 (registration number: CRD42024536459).

### Search Strategy for Study Identification

Search for original articles were performed within the Web of Science (Clarivate Analytics), Scopus (Elsevier), SciELO (Scientific Electronic Library Online), and PubMed (NLM) databases, published between January 1, 2014 and April 13, 2024. Our search strategy focused on Boolean connections (AND, OR) and the following keywords were used: “health literacy” (*alfabetización en salud*) and “self-management” (*autogestión*) OR “self-care” (*autocuidado*). The terms “self-care” and “self-management” are used interchangeably throughout, whereby self-management is understood as a point in the wider self-care continuum, as suggested by previous studies.^41^^,^^42^ The languages used for the search were Spanish and English. The last search was performed on April 13, 2024. Full search strategies per database are provided in Supplementary Material 2.

### Eligibility Criteria

Eligibility criteria were guided by the PICO format (population, interventions, comparators, and outcomes) recommended by the Cochrane Evidence Synthesis for Systematic Reviews.^43^

The population referred to patients diagnosed with a chronic disease >18 years. Intervention referred to HL interventions delivered in patients, aiming to strengthen, support or increase SM, as operationalized according to Nutbeam’s Tripartite Model.^25^ Study design included randomized controlled trials (RCTs) and quasi-experimental studies. Comparators included no treatment control groups, attention control groups (participants receive some other attention), or standard care control groups. Outcomes were SM or one of the domains of SM. Only studies published in English and Spanish peer-reviewed journals with full text available were included. Publication dates of studies were from 2014 to 2024.

Studies were excluded if they were protocols, systematic reviews, cross-sectional designs, or qualitative research. In addition, studies targeted to children and adolescents with a chronic disease were excluded. Interventions that did not consider at least one of the SM domains in their measurement were excluded.

### Selection of Sources of Evidence

The web-based tool Rayyan (Rayyan Systems Inc) was used to facilitate blinded review.^44^^,^^45^ First, duplicate records obtained from the databases were eliminated. Subsequently, 2 reviewers (M.-F.C. and G.N.) independently selected the records that met the inclusion criteria by reading the title and abstract of the articles. Articles that met the inclusion criteria were screened full text. If reviewers were unable to determine whether a study met inclusion criteria based on initial title and abstract review, the article was also included for full-text review. To determine interrater reliability, a calibration exercise was performed by pilot testing a random sample of 100 citations. A kappa of 0.78 was achieved. Discrepancies were resolved by discussion and obtaining consensus between 2 reviewers (M.-F.C. and G.N.) and by a third independent reviewer (J.C.).

### Critical Appraisal of Individual Sources of Evidence

The risk of bias was assessed using the Joanna Briggs Institute (JBI) standardized appraisal tools to evaluate safeguards for study validity and quality.^46–48^ For the RCTs, the JBI checklist for RCTs was employed.^46^ This 13-item checklist covers domains such as selection and allocation bias (items 1-3), intervention/exposure bias (items 4-6), outcome assessment and measurement bias (items 7-9), participant retention bias (item 10), and statistical validity (items 11-13). Each item was rated as yes, no, unclear, or not applicable. If “yes” is answered, a point is obtained. Then, studies were categorized as: high risk of bias (≤6 affirmative responses), moderate risk of bias (7-9 affirmative responses), and low risk of bias (≥10 affirmative responses).

In the quasiexperimental studies, JBI checklist for quasi-experimental studies was used.^47^ This 9-item checklist covers domains such as temporal procedure bias (item 1), selection and allocation bias (item 2), confounding factors bias (item 3), administration of intervention/exposure bias (item 4), outcome assessment, detection and measurement bias (item 5-7), participant retention bias (item 8), and statistical validity (item 9). If “yes” is chosen for the item, a point is obtained. Then, studies were categorized as: high risk of bias (≤4 affirmative responses), moderate risk of bias (5-7 affirmative responses), and low risk of bias (≥8 affirmative responses). In both quasi-experimental studies and RCTs, a study considered to have a low risk of bias demonstrates a high level of methodological rigor, with minimal bias and high reliability and validity of the results.

Two reviewers (M.-F.C. and G.N.) evaluated the risk of bias, with discrepancies resolved by discussion and obtaining consensus between 2 reviewers and by a third independent reviewer (J.C.). All articles were included regardless of the score obtained on the checklists.

### Synthesis of Results

The collected data were collated and analyzed, and results and outcomes were reported in a descriptive numerical summary and tabulation of findings. Data extraction covered studies’ general characteristics, intervention components, theoretical models of HL or SM, HL and SM definitions, HL and SM instruments, and effectiveness. Intervention components were analyzed in terms of modality (face-to-face or app- or web-based), duration (length of the intervention), content (topics covered), and delivery strategies (interactive resources, practical exercises, discussions, posters, and/or patient testimonials).^31^ A Synthesis Without Meta-analysis (SWiM) checklist is provided in Supplementary Material 3.

### Effectiveness Summary

Results are presented using a narrative synthesis, as the large number and heterogeneity of outcome measures rendered a meta-analysis unsuitable. We created the following scoring system to indicate effectiveness: Significant effects are effects where there was an improvement in SM outcomes, with a *P*-value of <.05. The between-group effect size was calculated using Cohen’s 𝑑, where the difference in mean change between the intervention and control groups was divided by the pooled standard deviation. In designs without a control group, effect size was calculated based on the pre-post intervention mean difference. The same method was used to establish the effectiveness on indirect effects, defined as effects on clinical outcomes or biomarkers. Effect sizes up to 0.49 were considered small, between 0.50 and <0.80 medium, and ≥0.80 large.^49^ In cases where only the median and interquartile range were available, the mean and standard deviation were estimated following the method proposed by Wan et al.^50^  *A priori* and *post hoc* power analyses were reported when documented in the studies.

## Results

### Selection of Sources of Evidence

**Figure 1.**
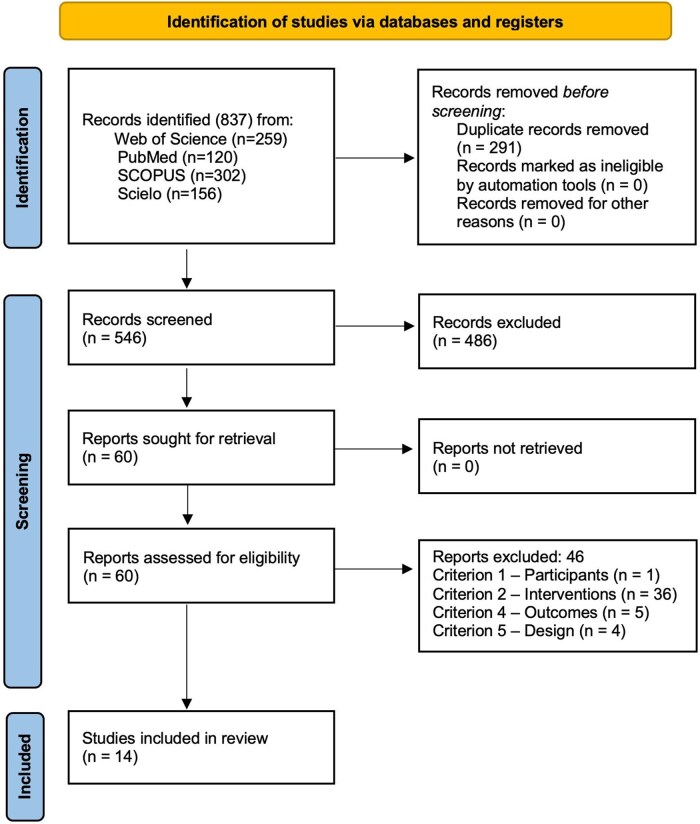
Preferred reporting items for systematic reviews and meta-analyses (PRISMA 2020) of included articles relating to health literacy interventions.^39^

The database search identified 837 potentially relevant documents (PubMed [*n* = 120], ScIELO [*n* = 156], SCOPUS [*n* = 302], and Web of Science [*n* = 259]). Following duplicate removal, 546 articles were screened on title and abstract, resulting in 60 potentially eligible full-text articles based on study population, outcome measure, study design, and type of intervention. A further 46 studies were excluded based on review of the full text (details in Supplementary Material 4). A total of 14 studies were included in this review. The outcomes of the study selection process were presented in a flow chart (Figure 1).

### Characteristics of Articles and Interventions Included

Six studies employed quasi-experimental designs [ID: 3,4,5,6,7,10], and 8 were RCTs [ID: 1,2,8,9,11,12,13,14]. All studies were published between 2014 and 2024. Studies were conducted across a range of countries and settings, including Taiwan (*n* = 3), Iran (*n* = 3), and one study each in the United States, Norway, Brazil, Canada, South Korea, Thailand, ­ Turkey, and Mexico. Studies were implemented in hospitals (*n* = 8), public primary healthcare (*n* = 4), community settings (*n* = 1), and home-based care (*n* = 1). The study population exhibited a high level of homogeneity, with nearly all studies encompassing patients with type 2 diabetes mellitus (T2DM) (*n* = 10). The remaining 4 studies focused on chronic obstructive pulmonary disease, heart failure, ischemic heart disease, or rheumatoid arthritis. A summary of all included studies and the characterization of interventions is presented in Supplementary Material 5.

### Risk of Bias

The risk of bias assessment using the JBI instrument is given in Supplementary Material 6. Four studies were found to have a low risk of bias, 9 a moderate, and 1 a high risk of bias. Nearly all studies employed adequate random sequence generation, allocation concealment, and outcome measurement. The main sources of bias were participant awareness of assigned interventions (*n* = 7), lack of blinding among intervention providers (*n* = 6), and statistical analysis concerns (*n* = 5).

Among the 13 effective interventions, only 4 had a low risk of bias. The unsuccessful intervention showed a moderate risk of bias. The study with the highest risk of bias showed the largest effect size on SM, with concerns about the lack of blinding for the outcome assessor, treatment deliverers, and participants. The person who implemented the intervention also gathered the study data [ID: 6]. Studies with >70 participants per condition also showed moderate risk of bias, with concerns regarding blinding procedures [ID: 8,9].

### Effect of HL Interventions on SM

Effect sizes on the total SM score were calculated in 10 of the 14 studies (Table 1), of which 9 studies showed a significant intervention effect, ranging between 0.38 to 7.35 [ID: 2,14]. Four interventions were reported as being effective by the authors but did not provide sufficient information to calculate the effect size [ID: 7,8,10,13]. In total, 13 studies reported an improved SM total score. Of the 14 studies, 10 documented a power analysis, with 9 studies achieving a sample size resulting in a power (1 − β) of ≥0.80. Two studies included more than 70 participants per group [ID: 8,9].

**Table 1.** Effect of health literacy interventions on self-management and clinical outcomes.

ID	Author	Disease	SM or self-care total score^a^	Medical management	Emotional management	Role Management	Clinical Outcomes
1	Ağralı & Akyar.^51^	T2DM	Improved SM (d = 0.71, 95% CI, 0.34-1.08) At 6 months (I-C)	Improved self-efficacy^b^ (*d* = 0.71, 95% CI, 0.34-1.08)	-	-	No change in HbA1c (*d* = 0.18, 95% CI, −0.17-0.54)
2	Borge et al.^52^	Chronic obstructive pulmonary disease	Improved SM (*d* = 0.38, 95% CI, −0.5-−0.1) At 6 months (I-C)	No change in disease knowledge (*d* = −0.22, 95% CI, −0.5- 0.04)	-	No change in navigation healthcare system (*d* = 0.13, 95% CI, −0.4-0.01)	Improved hospitalization rate (β = 1.40, 95% CI, 0.70-2.9)
3	Han et al.^53^	T2DM	Improved self-care (*d* = 0.50, 95% CI, −0.34-1.34) At 3 months (I1-I2)	Improved diet^c^ Improved physical activity^c^ Improved medication adherence^c^ Improved self-efficacy^c^ (*d* = 0.64, 95% CI, −0.21-1.49) Improved disease knowledge (*d* = 0.66, 95% CI, −0.19-1.52)	-	-	Improved HbA1c (*d* = −0.39, 95% CI, −0.44-1.23) Improved blood pressure (*d* = −0.16, 95% CI, −1.00-0.67) Improved FPG (*d* = −0.09, 95% CI, −0.74-0.92)
4	Barkhordari-Sharifabad et al.^54^	Heart failure	Improved self-care (*d* = 1.50, 95% CI, 0.91-2.09) At 1 month (I-C)	Improved diet^c^ Improved physical activity^c^ Improved self-efficacy (*d* = 0.84, 95% CI, 0.64-1.04)	-	-	-
5	Guo et al.^55^	T2DM	No change in self-care (*d* = −0.28, 95% CI, −0.66-0.10) At 3 months (I-C)	No change in diet (*d* = −0.11, 95% CI, −0.53-0.30)	-	-	Improved HbA1c (*d* = 0.016, 95% CI, −0.40-0.43)
6	Hakimzadeh & AdibHajbaghery.^56^	Ischaemic heart disease	Improved self-care (*d* = 7.19, 95% CI, 5.68-8.71) At 3 weeks (I-C)	Improved diet (*d* = 4.72, 95% CI, 3.64-5.80) No change in physical activity (*d* = 1.00, 95% CI, 0.41-1.59) Improved medication adherence (*d* = 11.5, 95% CI, 9.17-13.82)	Improved response to stressful situations (*d* = 5.15, 95% CI, 4.00-6.30)	-	-
7	Hung et al.^57^	T2DM	Improved self-care^c^ At 3 months (I-C)	Improved SMBG (β = 1.97, SE: 0.57, *P* < .01) Improved smoking (*d* = 6.45, 95% CI, 5.07-7.84) Improved disease knowledge (β = 3.22, SE = 1.32, *P* < .05)	-	-	Improved HbA1c (β = 0.70, SE: 0.25, *P* < .05) Improved FPG (β = −46.93, SE: 11.98, *P* < .01) Improved BMI (kg/m^2^) (β = −1.73, SE: 0.41, *P* < .01)
8	Kim et al.^58^	T2DM	Improved self-care^c^ At 3 months (I-C)	Improved diet^c^ Improved physical activity^c^ Improved SMBG^c^ Improved medication adherence^c^ Improved self-efficacy (*d* = 0.785 95% CI, 0.50-1.06) Improved disease knowledge (*d* = 0.691 95% CI, 0.41-0.97)	-	-	Improved HbA1c (β = −0.195, 95% CI, 0.46-0.07) No change in blood pressure (*d* = −0.066, 95% CI, 0.33-0.20) Improved cholesterol (β = −0.305, 95% CI, 0.57-0.03) Improved triglyceride (β = −0.199, 95% CI, 0.47-0.07)
9	Leong et al.^59^	T2DM	Improved self-care (β = 0.073, 95% CI, −0.21-0.36) At 3 months (I-C)	No change in diet (*d* = 0.092, 95% CI, −0.19-0.38) No change in physical activity (*d* = 0.054, 95% CI, −0.23-0.34) Improved medication adherence (*d* = 0.269, 95% CI, 0.02-0.56) Improved disease knowledge (*d* = 0.287, 95% CI, 0.004-0.58)	-	-	No change in HbA1c **(** *d* = 0.37, 95% CI, 0.07-0.66)
10	Moura et al.^60^	T2DM	Improved self-care^c^ At 1 month (I1-I2)	Improved diet^c^ Improved physical activity^c^ Improved SMBG^c^ Improved medication adherence^c^ No change in smoking^c^	-	-	-
11	Michou et al.^61^	Rheumatoid arthritis	Improved self-care (*d* = 0.52, 95% CI, 0.14-0.89) At 3 months (I-C)	No change in medication adherence (*d* = 0.02, 95% CI, −0.34-0.39)	-	-	-
12	Sriklo et al.^62^	T2DM	Improved SM (*d* = 0.60, 95% CI, 1.93-3.27) At 2 months (I-C)	Improved diet^c^ Improved physical activity^c^ Improved medication adherence^c^	Improved stress management^c^	-	Improved HbA1c (*d* = −0.13, 95% CI, −1.88-−0.80)
13	Whittemore et al.^63^	T2DM	Improved SM^c^ At 6 months (I-C)	No change in diet (*d* = 0.34, 95% CI, −0.23-0.92) No change in physical activity (*d* = −0.19, 95% CI, 0.76-0.38) Improved SMBG (*d* = 2.07, 95% CI, 1.36-2.78) Improved self-efficacy (*d* = 0.60, 95% CI, 0.60-1.19)	-	-	Improved HbA1c (*d* = 0.13, 95% CI, −0.44-0.70) No change in blood pressure (*d* = −0.04, 95% CI, 0.61-0.53)
14	Zeidi et al.^64^	T2DM	Improved self-care (*d* = 7.35, 95% CI, 6.50-8.19) At 2 months (I-C)	Improved diet (*d* = 1.95, 95% CI, 1.95-2.32) Improved physical activity (*d* = 2.70, 95% CI, 2.28-3.13) Improved SMBG (*d* = 2.60, 95% CI, 2.19-3.02) Improved medication adherence (*d* = 5.16, 95% CI, 4.53-5.79)	-	-	-

. Abbreviations: -, not applicable; β, beta coefficient; BMI, body mass index; BP, systolic blood pressure; CI, confidence interval 95%; FPG, fasting plasma glucose; HbA1c, glycated hemoglobin; SM, self-management; SMBG, self-monitoring of blood glucose; T2DM, type 2 diabetes mellitus

a SM and self-care are used interchangeably according to previous studies.

b It refers to self-efficacy in disease management.

c Studies reported improvements in the variable (mean differences) but did not provide sufficient data to calculate the effect size (standard deviation or standard error was not delivered). I1-I2: effect sizes were calculated based on the mean differences and standard deviations (or standard error) preintervention (T1) and postintervention (T2) in studies without a control group. I-C: effect sizes were calculated based on the mean differences and standard deviations (or standard error) between the intervention (I) and control (C) groups after HL intervention. Cohen’s *d* = effect sizes up to 0.49 small, between 0.50 and <0.80 medium, and ≥0.80 large. *B*: It is a regression coefficient that gained from GEE analysis.

#### Effectiveness on Medical Management

Fourteen studies addressed the Medical Management domain, covering treatment adherence (medication: *n* = 8; blood glucose self-monitoring: *n* = 5), lifestyle behaviors (e.g., physical activity, diet, smoking; *n* = 11), and attitudes and knowledge (*n* = 10). Effect sizes were calculated for 12 studies, 9 of which showed a significant HL intervention effect in at least 1 component. Two additional studies were described as effective by the authors but lacked data to calculate effect sizes [ID: 10,12]. In total, 11 studies reported improvements in medical SM.

A detailed analysis revealed that 3 out of 4 studies reported significant improvements in medication adherence, with effect sizes ranging from 0.26 to 11.50 [ID: 6,9]. Similarly, all 3 studies examining blood glucose self-monitoring demonstrated significant effects (1.97–2.60 [ID: 7,14]).

Findings related to lifestyle behaviors were mixed. Significant effects on diet were observed in 2 of 5 studies (1.95–4.72 [ID: 6,14]), while only one of 4 studies found a significant effect on physical activity (2.70 [ID: 14]). Of the 2 studies evaluating smoking, one reported a significant effect (6.45 [ID: 7,10]).

Regarding attitudes and knowledge, 5 studies reported significant improvements in self-efficacy (0.60–0.84 [ID: 4,13]), and 4 out of 5 studies showed significant effects on disease knowledge (0.28-0.69 [ID: 8,9]).

#### Effectiveness on Emotional Management

Two studies examined the emotional management domain. One study showed a significant effect on emotional management, with an effect size of 5.15 [ID: 6]. Another study also improved emotional management but did not provide sufficient data to calculate the effect size [ID: 12].

#### Effectiveness on Role Management

Only one study examined the role management domain [ID: 2]. This study did not find a significant effect of the HL intervention on role management.

#### Indirect Effect on Clinical Outcomes

In 9 studies, the effect on several clinical outcomes was measured: HbA1c (*n* = 8), blood pressure (*n* = 3), fasting plasma glucose (*n* = 2), body mass index (BMI) (*n* = 1), hospitalization rates (*n* = 1), cholesterol (*n* = 1), and triglyceride levels (*n* = 1). Overall, small effect sizes of HL interventions on clinical outcomes were observed (0.016-0.39 [ID: 5,3]). Three studies reported results using β values. The highest β value was for BMI (β = −46.93) and the lowest for HbA1c (β = −0.195). One study assessed hospitalization rates and found a significant effect (β = 1.40).

### Theoretical Foundations and Conceptualization (definitions and measures)

The studies varied in their theoretical foundations and conceptualizations. Nine out of the 14 studies identified theoretical foundations [ID: 1,2,3,5,6,8,12,13,14], including the Health Belief Model^65^ [ID: 1], Motivational Interviewing^66^ [ID: 2], von Wagner’s model,^67^ and the Theory of Planned Behaviours^68^ [ID: 11,14]. Other studies were based on the Transformative Learning Model^69^ [ID: 12] and Health Action Process Approach^70^ [ID: 13]. Five studies did not specify a theoretical foundation.

Definitions of HL also differed. Eight of the 14 studies clearly articulated a definition of HL, drawing on preexisting frameworks from the literature [ID: 1,2,3,5,6,10,12,14]. Most frequent definitions used were those proposed by Nutbeam^26^ (*n* = 2) and the World Health Organization^71^ (*n* = 2). These definitions emphasized the skill needed to use health information for decision-making, including cognitive and social abilities for access, comprehension, and application. Five definitions did not address the critical dimension of HL. Nutbeam’s definition was the only one that included critical HL.

Health literacy measures were self-administered. Four studies evaluated disease-specific knowledge [ID: 3,7,8,10], 7 used generic HL tools [ID: 1,2,5,9,11,12,14], and 1 created a measure specifically for the study [ID: 7]. The most frequently used measure (*n* = 2) was the HL Scale.^72^ Regarding the components of HL (functional, communicative, critical), all instruments measured functional HL (e.g., numeracy skills and reading comprehension), 4 measured communicative HL [ID: 1,2,11,12], and 2 measured critical HL [ID: 1,12].

Only one study [ID: 12] explicitly defined SM. This definition emphasized the responsibility for managing care during chronic disease, including medical, role, and emotional aspects.^10^ The definition was based on preexisting frameworks from the literature and included key components such as symptom management, seeking social support, and coping strategies to manage psychological challenges associated with chronic illness.

All SM instruments were self-administered. Nine studies evaluated disease-specific knowledge [ID: 1,3,4,5,7,8,9,10,13], 6 used generic SM tools [ID: 2,6,11,12,13,14], and 1 created a measure specifically for the study [ID: 3]. The most frequently used measure (*n* = 3) was the Summary of Diabetes Self-Care Activities Questionnaire.^73^ Regarding the components of SM, in all studies medical SM was measured, including actions undertaken to maintain a healthy lifestyle and managing medical aspects of chronic diseases. In only 3 studies, role and emotional management was measured. Furthermore, 1 study utilized a self-efficacy instrument to measure SM [ID: 1]. A summary of the theoretical foundations and conceptualization of HL and SM is presented in Supplementary Material 7.

### Intervention Components

Interventions were analyzed based on 4 topics: modality, duration, content, and delivery strategies. Ten studies were group-based face-to-face interventions [ID: 1,2,3,6,7,8,10,12,13,14], and 4 were app- or web-based interventions [ID: 4,5,9,11].

The duration time of the interventions varied from a 1-time 30-minute video to 7-weekly sessions with daily text/picture messages delivered over 6 months. Eight studies carried out follow-up periods ranging from 3 months to 1 year, with most lasting more than 3 months.

In relation to the content, most interventions focused on disease management and adherence to diet and physical activity [ID: 1,2,3,6,7,8,9,10,11,13,14]. Four interventions included strategies for preventing or managing depression or promoting well-being; however, their evaluation focused on depressive symptoms rather than stress management [ID: 3,4,11,13]. Only 2 studies included problem-solving in their content [ID: 4,8].

Most studies used a combination of strategies to implement the intervention. For example, 10 studies incorporated interactive videos with multimedia, images, role-playing, Q&A forums, and quizzes [ID: 1,2,3,4,5,7,8,9,12,11]. In one case, a health education teleconference was designed [ID: 11]. Four studies demonstrated practical activities, such as washing, drying, and hydrating one’s own feet, to illustrate proper techniques for diabetes self-care [ID: 3,5,10,14]. Three interventions used a roundtable discussion with educational posters or a booklet with illustrations to visualize the disease’s effects on the body and the importance of control [ID: 1,6,11]. One study included patient testimonials to reinforce adherence [ID: 11]. Three studies incorporated tailored or culture-specific elements into their interventions [ID: 3,8,13]. Tailoring involved adapting educational materials to align with an individual’s cultural background and language proficiency (e.g., minority groups). The components of the interventions are reported in Supplementary Material 5.

## Discussion

This systematic review examined the effectiveness of HL interventions on SM in patients with chronic diseases. Of the 14 studies included, 13 demonstrated a significant effect on SM total score, with effect sizes ranging from 0.38 to 7.35. In 11 studies HL interventions were effective in improving medical SM, with the largest effect on medication adherence and self-monitoring. However, within the domain of medical SM, the effectiveness on diet and physical activity was unclear. Two studies were effective to enhance emotional management. No study was effective on role management. Theories used to define HL and SM as well as the measurements of HL and SM showed incongruence. In terms of the components of the interventions, most studies employed a combination of implementation strategies, with face-to-face formats being predominant. The risk of bias was moderate, particularly concerning blinding of interventions.

Most HL interventions in the reviewed research were effective in increasing SM. Studies with low to moderate risk of bias showed significant effects of HL interventions on 3 components of medical management: medication adherence, disease knowledge, and self-efficacy in disease management, with effect sizes ranging from small to large. Some studies were characterized as effective but lacked sufficient data for calculating effect sizes. Consistent with prior evidence, a positive trend toward the effect of HL interventions on SM is found in disease-specific reviews in chronic conditions such as chronic obstructive pulmonary disease,^33^ diabetes,^58^ heart failure,^74^ as well as in healthy populations.^75^ Similarly, a recent systematic review of HL interventions reported a statistically significant effect post-intervention in 72% of the studies.^31^ Whereas most studies focus on total SM scores, our study offers a robust and differentiated picture of the efficacy of these interventions by estimating effect sizes at a domain-specific level.

However, the effects on adherence to diet and exercise were inconclusive. This aligns with previous research, which has observed that patients are often reluctant to adopt healthy lifestyle changes due to factors such as stress, past failed attempts, or satisfaction with their current lifestyle.^76^^,^^77^ Thus, patients are generally more adherent to medications than to lifestyle changes.^78–80^ Another explanation is that while HL interventions may enhance disease knowledge, knowledge alone may not be enough to drive behavioral change. Jager et al^81^ highlight that individuals with low HL experience a persistent gap between awareness and the ability to translate that knowledge into action. The limited effectiveness on diet and physical activity reflects the need for additional strategies to transform health knowledge into concrete changes in diet and exercise, for example, by enhancing action planning and patient motivation.

Despite recognizing role and emotional management as essential for effective SM,^10^^,^^82^ only 3 studies addressed these domains. Most studies focused on medical management, with SM instruments primarily assessing medical aspects. This suggests that psychosocial factors may be underrepresented. A systematic review of 31 studies on chronic obstructive pulmonary disease SM concluded that the manner in which patients manage their emotions can influence the ways in which they perform their role adjustment and medical management.^83^ For instance, individuals who manage their emotions effectively are more likely to articulate their needs and seek support proactively.^21^^,^^82^^,^^84^ These results suggest the importance of integrating emotional strategies into HL interventions.

Regarding indirect effects most studies examined controlling HbA1C. We found a small but significant effect size of HL interventions on clinical outcomes (ie, HbA1c, BMI, blood pressure, cholesterol, triglyceride, and hospitalization rate) for studies with low to moderate risk of bias. Similar to our study, other studies have shown that HL interventions have no or only a small effect on physiological indicators such as blood pressure and lipid levels.^85^^,^^86^ Although the reduction in HbA1c levels is commonly used to measure whether diabetes interventions are successful, from a behavioral change intervention perspective, it is possible that biomarkers do not fully capture the complexity of health behaviors.^87^ For example, behavior changes may not readily translate into outcome indicators like BMI. To reflect the global impact of interventions, a comprehensive evaluation of effectiveness must include both clinical and behavioral outcomes.^85^

Inconsistency between HL definitions and the HL instruments used was observed in almost all studies. Despite including broad definitions of HL (functional, communicative, and critical HL), some of the included studies only focused on reading and numeracy skills (functional literacy) in the measured outcome [ID: 3,5,10]. Additionally, few studies provided clear definitions of SM on which the measurements were based. In some cases, even when measurements were designed to assess SM, closer examination revealed that they measured related constructs, such as self-efficacy or skill acquisition. These findings are consistent with prior research.^88–90^ The discrepancy between definitions and measures can impact the effectiveness of HL interventions, having implications for the examination of the hypothesis of causality. Thus, it remains critical to determine how to assess the effectiveness if it is not clear what the interventions are supposed to accomplish.^31^ Theory-based interventions, methodological consistency, and comprehensive HL and SM measures are required to determine optimal strategies for strengthening SM through HL interventions.

The reviewed interventions typically lasted 1-12 months and focused on disease knowledge, adherence, and lifestyle changes. We identified variability in intervention timing, content, and modalities, which prevented us from examining which format or components were most effective. However, prior research suggests that effective strategies to optimize patient SM include simplifying language, visual aids, and techniques such as the teach-back method to identify misunderstandings.^91^ Furthermore, individualized approaches appear more effective for individuals with low HL.^92^ Beyond individual-level factors, the success of HL interventions also depends on organizational culture and commitment.^93^

Interestingly, using modern technology as a primary intervention mode was limited, with only 4 out of 14 studies incorporating web-based approaches. This contrasts with previous reviews of HL interventions, which found the web mode dominant.^75^^,^^94^ In our review, the only study that did not find a significant effect on SM was the one conducted through a mobile eHL application. Similarly, other remote interventions using websites, messages, or interactive software have not been effective in improving HL.^95–98^ One possible explanation is that mobile eHL programs aim to empower patients rather than directly modify undesirable behaviors.^98–100^ Furthermore, patients with chronic diseases are generally older and might not have the skills to use e-health interventions appropriately.^101–103^ More research is needed to understand how to integrate technology-based approaches into comprehensive healthcare management.

Our findings should be interpreted with caution, as most studies had a moderate risk of bias. Despite generally strong ratings for study design and data collection, concerns arose regarding selection bias, blinding, statistical analysis, and/or lack of follow-up. Our results suggest that blinding was the primary concern, even in studies with higher statistical power and larger sample sizes. Blinding is crucial for internal validity in RCTs,^104^ but it may not always be feasible for HL interventions. In the studies with blinding limitations, we generally observed small to moderate effect sizes on SM domains immediately after intervention, except for one study, which presented an exceptionally large effect size. This study also had the highest risk of bias, compounded by unreliable measurements, further raising concerns about the validity of its results.

The reviewed studies presented several limitations. First, most interventions were short-term, typically lasting less than 3 months, which limited their potential to generate long-lasting behavioral change. Second, the studies lacked comprehensive cost-effectiveness analyses, which are vital for determining the financial feasibility and broader applicability of HL interventions within healthcare systems.^105^ Third, there was a predominant focus on type 2 diabetes, with insufficient attention given to multimorbidity and other prevalent chronic diseases, such as chronic kidney disease and hypertension, which remain less explored in the literature.^31^^,^^106^

### Strengths and Limitations

A major strength of this review is the inclusion of studies from 10 countries with over 1000 participants with chronic diseases, providing a balanced representation of interventions from both developed and developing countries. Moreover, we used a variety of checklists to assess the methodological quality of the systematic review. The study selection, data extraction, and quality appraisal were structured in accordance with PRISMA guidelines. However, certain limitations of the systematic review itself need to be acknowledged. First, the moderate risk of bias of the included studies restricted the ability to draw definitive conclusions on intervention effectiveness. Second, the search strategy focused on “HL interventions” may have been less sensitive to studies targeting SM in populations with limited HL that were not labelled as such (e.g., health education). Notwithstanding this, since we provide an extensive overview, it is our opinion that such additional evidence would not greatly affect our conclusions. Additionally, our decision to limit the review to studies published after 2014 hindered a more comprehensive historical analysis of HL and SM literature. Despite these limitations, our review included studies from diverse contexts, highlighting the importance of cultural sensitivity in implementing HL interventions.^81^^,^^107–109^

### Implications for Research and Practice

The variety of effective interventions in this review provides the opportunity to offer a range of HL interventions for increasing SM in chronic diseases. Our findings provide substantial evidence supporting the implementation of interventions focused on medical management in clinical practice. However, clinicians should work to address the psychological ramifications of chronic conditions, incorporating emotional and role management within the conceptual framework of SM theory.^10^^,^^21^^,^^110^ Therefore, more attention should be given to interventions focusing upon role and emotional management.

To advance research on HL interventions aimed at enhancing evidence-based practice, it is necessary to establish an approach with methodological coherence that articulates theoretical frameworks, clearly defined components, and appropriate evaluation instruments. Thus, HL interventions should be operationalized and measured consistently, to rigorously evaluate program effectiveness. Moreover, following the tripartite model of HL proposed by Nutbeam,^26^ which emphasizes functional, interactive, and critical elements, it is expected that HL definitions and interventions should address these 3 domains.

Health literacy interventions must be culturally appropriate and group-specific, while being person-centered. For example, patients can be stratified based on their HL level.^38^ Since HL is, in part, culturally conditioned, it is crucial to conduct studies in diverse cultural contexts. It is hypothesized that findings from high-income countries with highly educated populations may not be transferable to low-income countries, where health systems are often uncoordinated and segmented, potentially negatively impacting the people they serve.^110^^,^^111^ Future interventions should combine cultural responsiveness and tailored approaches to increase their effectiveness.

Future studies must also address the cost-effectiveness and feasibility of interventions in routine care, as these are essential for their large-scale implementation within healthcare systems. Additionally, further research should involve larger, more geographically diverse studies with extended follow-up to assess long-term effects. Behavioral change requires time, guidance, and sustained effort^81^; therefore, it is crucial to provide support to patients to maintain the desired behaviors beyond the initial implementation of the program. Improving the rigor of the methods used is also important, such as addressing bias, controlling confounders, and utilizing blinding procedures where applicable.

## Conclusions

Health literacy interventions have proven effective in improving SM in patients with chronic diseases by strengthening patients’ understanding of their condition and providing them with relevant techniques. Nonetheless, heterogeneity in terms of clinical (ie, patient groups, intervention characteristics) and methodological (ie, study design, outcome measurement) features have hindered the comparison and synthesis of findings, limiting the development of generalizable conclusions. To better understand the effect of HL on SM, theory-based interventions, methodological consistency, and comprehensive HL and SM measures are needed. Future interventions should prioritize role and emotional management and promote a person-centered approach.

## Supplementary Material

kaaf073_Supplementary_Data

## Data Availability

All data collected are presented in the main manuscript and Supplementary Material.

## Open science transparency statements

1. Study registration: The study was preregistered at PROSPERO (https://www.crd.york.ac.uk/PROSPERO/view/CRD42024536459).
2. Analytic plan preregistration: The analysis plan was not formally preregistered.
3. Analytic code availability: Study quality was assessed using the “Quality Assessment Tool for RCT and Quasi-experimental Studies” developed by the Joanna Briggs Institute (JBI). Effect sizes within and between groups were calculated using the URL: https://www.campbellcollaboration.org/calculator/
4. Materials availability: The included studies in this review are publicly available via PubMed.
