# Supplementary material for: The Effect of Health Literacy Interventions on Self-management in Chronic Diseases: A Systematic Review
Source: Ann Behav Med. 2025 Oct 31;59(1):kaaf073. doi: 10.1093/abm/kaaf073 (PMC12579557; doi:10.1093/abm/kaaf073)
Supplement: kaaf073_Supplementary_Data [file kaaf073_supplementary_data.docx]

**Electronic Supplementary Materials [Tables 1-7]**

**Table SM1.** PRISMA 2020 Checklist

| **Section and Topic** | **Item #** | **Checklist item** | **Location where item is reported** |
| --- | --- | --- | --- |
| **TITLE** | | |  |
| Title | 1 | Identify the report as a systematic review. | **Title Page** |
| **ABSTRACT** | | |  |
| Abstract | 2 | See the PRISMA 2020 for Abstracts checklist. | **Abstract** |
| **INTRODUCTION** | | |  |
| Rationale | 3 | Describe the rationale for the review in the context of existing knowledge. | **Introduction** |
| Objectives | 4 | Provide an explicit statement of the objective(s) or question(s) the review addresses. | **Introduction** |
| **METHODS** | | |  |
| Eligibility criteria | 5 | Specify the inclusion and exclusion criteria for the review and how studies were grouped for the syntheses. | **Eligibility Criteria** |
| Information sources | 6 | Specify all databases, registers, websites, organisations, reference lists and other sources searched or consulted to identify studies. Specify the date when each source was last searched or consulted. | **Approach and Review Registration** |
| Search strategy | 7 | Present the full search strategies for all databases, registers and websites, including any filters and limits used. | **Search strategy and SM2** |
| Selection process | 8 | Specify the methods used to decide whether a study met the inclusion criteria of the review, including how many reviewers screened each record and each report retrieved, whether they worked independently, and if applicable, details of automation tools used in the process. | **Selection of Sources of Evidence** |
| Data collection process | 9 | Specify the methods used to collect data from reports, including how many reviewers collected data from each report, whether they worked independently, any processes for obtaining or confirming data from study investigators, and if applicable, details of automation tools used in the process. | **Synthesis of the Results** |
| Data items | 10a | List and define all outcomes for which data were sought. Specify whether all results that were compatible with each outcome domain in each study were sought (e.g. for all measures, time points, analyses), and if not, the methods used to decide which results to collect. | **Synthesis of the Results** |
|  | 10b | List and define all other variables for which data were sought (e.g. participant and intervention characteristics, funding sources). Describe any assumptions made about any missing or unclear information. | **Synthesis of the Results** |
| Study risk of bias assessment | 11 | Specify the methods used to assess risk of bias in the included studies, including details of the tool(s) used, how many reviewers assessed each study and whether they worked independently, and if applicable, details of automation tools used in the process. | **Risk of Bias** |
| Effect measures | 12 | Specify for each outcome the effect measure(s) (e.g. risk ratio, mean difference) used in the synthesis or presentation of results. | **Effectiveness Summary** |
| Synthesis methods | 13a | Describe the processes used to decide which studies were eligible for each synthesis (e.g. tabulating the study intervention characteristics and comparing against the planned groups for each synthesis (item #5)). | **Effectiveness Summary** |
|  | 13b | Describe any methods required to prepare the data for presentation or synthesis, such as handling of missing summary statistics, or data conversions. | **Effectiveness Summary** |
|  | 13c | Describe any methods used to tabulate or visually display results of individual studies and syntheses. | **Effectiveness Summary** |
|  | 13d | Describe any methods used to synthesize results and provide a rationale for the choice(s). If meta-analysis was performed, describe the model(s), method(s) to identify the presence and extent of statistical heterogeneity, and software package(s) used. | **Effectiveness Summary** |
|  | 13e | Describe any methods used to explore possible causes of heterogeneity among study results (e.g. subgroup analysis, meta-regression). | **Not Applicable** |
|  | 13f | Describe any sensitivity analyses conducted to assess robustness of the synthesized results. | **Not Applicable** |
| Reporting bias assessment | 14 | Describe any methods used to assess risk of bias due to missing results in a synthesis (arising from reporting biases). | **Risk of Bias** |
| Certainty assessment | 15 | Describe any methods used to assess certainty (or confidence) in the body of evidence for an outcome. | **Risk of Bias + effect size when interpreting the results** |
| **RESULTS** | | |  |
| Study selection | 16a | Describe the results of the search and selection process, from the number of records identified in the search to the number of studies included in the review, ideally using a flow diagram. | **See Fig 1** |
|  | 16b | Cite studies that might appear to meet the inclusion criteria, but which were excluded, and explain why they were excluded. | **See SM4** |
| Study characteristics | 17 | Cite each included study and present its characteristics. | **See SM5** |
| Risk of bias in studies | 18 | Present assessments of risk of bias for each included study. | **See SM6** |
| Results of individual studies | 19 | For all outcomes, present, for each study: (a) summary statistics for each group (where appropriate) and (b) an effect estimates and its precision (e.g. confidence/credible interval), ideally using structured tables or plots. | **Results** |
| Results of syntheses | 20a | For each synthesis, briefly summarise the characteristics and risk of bias among contributing studies. | **Results** |
|  | 20b | Present results of all statistical syntheses conducted. If meta-analysis was done, present for each the summary estimate and its precision (e.g. confidence/credible interval) and measures of statistical heterogeneity. If comparing groups, describe the direction of the effect. | **Results** |
|  | 20c | Present results of all investigations of possible causes of heterogeneity among study results. | **Not Applicable** |
|  | 20d | Present results of all sensitivity analyses conducted to assess the robustness of the synthesized results. | **Not Applicable** |
| Reporting biases | 21 | Present assessments of risk of bias due to missing results (arising from reporting biases) for each synthesis assessed. | **Results** |
| Certainty of evidence | 22 | Present assessments of certainty (or confidence) in the body of evidence for each outcome assessed. | **Not Applicable** |
| **DISCUSSION** | | |  |
| Discussion | 23a | Provide a general interpretation of the results in the context of other evidence. | **Discussion** |
|  | 23b | Discuss any limitations of the evidence included in the review. | **Discussion** |
|  | 23c | Discuss any limitations of the review processes used. | **Discussion** |
|  | 23d | Discuss implications of the results for practice, policy, and future research. | **Discussion** |
| **OTHER INFORMATION** | | |  |
| Registration and protocol | 24a | Provide registration information for the review, including register name and registration number, or state that the review was not registered. | **Approach and Review Registration** |
|  | 24b | Indicate where the review protocol can be accessed, or state that a protocol was not prepared. | **Approach and Review Registration** |
|  | 24c | Describe and explain any amendments to information provided at registration or in the protocol. | **See Protocol** |
| Support | 25 | Describe sources of financial or non-financial support for the review, and the role of the funders or sponsors in the review. | **See Funding** |
| Competing interests | 26 | Declare any competing interests of review authors. | **See Conflict of Interest** |
| Availability of data, code and other materials | 27 | Report which of the following are publicly available and where they can be found: template data collection forms; data extracted from included studies; data used for all analyses; analytic code; any other materials used in the review. | **See Data Available** |

*From:* Page, M. J., McKenzie, J. E., Bossuyt, P. M., Boutron, I., Hoffmann, T. C., Mulrow, C. D., ... & Moher, D. (2021). The PRISMA 2020 statement: an updated guideline for reporting systematic reviews. *BMJ*, *372*. https://doi.org/10.1136/bmj.n71

**Table SM2.** Search strings for each WOS, Scopus, PubMed and Scielo database (n=837)

**2a**. Search strings for WOS data base

| *Syntaxis*: (TS=("health literacy") AND TS=("self-management" OR "self-care") NOT TS=("systematic review") NOT TS=("meta-analysis") NOT TS=("longitudinal stud*") NOT TS=("cohort stud*") NOT TS=("qualitative") AND TS=("random*" OR "experimental" OR "quasi-experimental")) AND (LA==("ENGLISH") AND PY==("2024" OR "2023" OR "2022" OR "2021" OR "2020" OR "2019" OR "2018" OR "2017" OR "2016" OR "2015"))   - Filters - Search phrase limited by title and abstract - Document type: articles - Language: English, Spanish, and Portuguese - Publication date: 2024-2014 - **Results: 259** |
| --- |

**2b.** Search strings for Scopus

| *Syntaxis*: ( TITLE-ABS-KEY ( "health literacy" ) AND TITLE-ABS-KEY ( "self-management" OR "self-care" ) AND TITLE-ABS-KEY ( "random*" OR "experiment" OR "quasi-experiment" ) AND NOT TITLE-ABS-KEY ( "systematic review" ) AND NOT TITLE-ABS-KEY ( "meta-analysis" ) AND NOT TITLE-ABS-KEY ( "cohort stud*" ) AND NOT TITLE-ABS-KEY ( "longitudinal stud*" ) AND NOT TITLE-ABS-KEY ( "qualitative" ) ) AND PUBYEAR > 2013 AND PUBYEAR < 2025 AND ( LIMIT-TO ( DOCTYPE , "ar" ) ) AND ( LIMIT-TO ( LANGUAGE , "English" ) OR LIMIT-TO ( LANGUAGE , "Portuguese" ) OR LIMIT-TO ( LANGUAGE , "Spanish" ) )   - Filters - Search phrase limited by title, abstract and keywords - Document type: articles - Language: English, Spanish, and Portuguese - Publication date: : 2024-2014 - **Results: 302** |
| --- |

**2c.** Search strings for PubMed

| *Syntaxis*: (("health literacy"[Title/Abstract] AND ("self-management"[Title/Abstract] OR "self-care"[Title/Abstract]) AND ("random*"[Title/Abstract] OR "experiment"[Title/Abstract] OR "quasi-experiment"[Title/Abstract])) NOT ("systematic review"[Title/Abstract] OR "meta-analysis"[Title/Abstract] OR "cohort stud*"[Title/Abstract] OR "longitudinal stud*"[Title/Abstract])) AND ((y_10[Filter]) AND (humans[Filter]) AND (English[Filter] OR Portuguese[Filter] OR Spanish[Filter]))   - Filters:   - Search phrase limited by title, abstract and keywords   - Document type: articles   - Language: English, Spanish, and Portuguese   - Publication date: 2024-2014 - **Results: 120** |
| --- |

**2d.** Search strings for Scielo

| *Syntaxis ENG*: (("health literacy") AND ("self-management" OR "self-care")) AND ("random*" OR "experimental" OR "quasi-experimental")   - Filters   - Search phrase limited by title, abstract and keywords   - Document type: articles   - Publication date: 2024-2014 - **Results: 2**   *Syntaxis ESP:* (((((((("alfabetizacion en salud") AND ("autogestion") OR ("auto cuidado")) AND ("random*") OR ("experimental") OR ("quasi-experimental")) AND NOT ("revisión sistemática") AND NOT ("meta-análisis"))) AND NOT ("longitudinal")) AND NOT ("cohorte")) AND NOT ("seguimiento")) AND NOT ("cualitativo")   - **Results: 154 + 2 = 156** |
| --- |

**Table SM3.** Synthesis Without Meta-analysis (SWiM) Reporting items

| **SWiM is intended to complement and be used as an extension to PRISMA** | | | |
| --- | --- | --- | --- |
| **SWiM reporting item** | **Item description** | **Page in manuscript where item is reported** | **Other*** |
| *Methods* | | | |
| **1** Grouping studies for synthesis | 1a) Provide a description of, and rationale for, the groups used in the synthesis (e.g., groupings of populations, interventions, outcomes, study design) | See page 8 |  |
|  | 1b) Detail and provide rationale for any changes made subsequent to the protocol in the groups used in the synthesis | Not Applicable |  |
| **2** Describe the standardised metric and transformation methods used | Describe the standardised metric for each outcome. Explain why the metric(s) was chosen, and describe any methods used to transform the intervention effects, as reported in the study, to the standardised metric, citing any methodological guidance consulted | See page 8 |  |
| **3** Describe the synthesis methods | Describe and justify the methods used to synthesise the effects for each outcome when it was not possible to undertake a meta-analysis of effect estimates | See page 8 |  |
| **4** Criteria used to prioritise results for summary and synthesis | Where applicable, provide the criteria used, with supporting justification, to select the particular studies, or a particular study, for the main synthesis or to draw conclusions from the synthesis (e.g., based on study design, risk of bias assessments, directness in relation to the review question) | See page 6 |  |
| **SWiM reporting item** | **Item description** | **Page in manuscript where item is reported** | **Other*** |
| **5** Investigation of heterogeneity in reported effects | State the method(s) used to examine heterogeneity in reported effects when it was not possible to undertake a meta-analysis of effect estimates and its extensions to investigate heterogeneity | Not Applicable |  |
| **6** Certainty of evidence | Describe the methods used to assess certainty of the synthesis findings | Not Applicable |  |
| **7** Data presentation methods | Describe the graphical and tabular methods used to present the effects (e.g., tables, forest plots, harvest plots).  Specify key study characteristics (e.g., study design, risk of bias) used to order the studies, in the text and any tables or graphs, clearly referencing the studies included | See Table 1 |  |
| *Results* | | | |
| **8** Reporting results | For each comparison and outcome, provide a description of the synthesised findings, and the certainty of the findings. Describe the result in language that is consistent with the question the synthesis addresses, and indicate which studies contribute to the synthesis. | See pages 9-15 |  |
| *Discussion* |  |  |  |
| **9** Limitations of the synthesis | Report the limitations of the synthesis methods used and/or the groupings used in the synthesis, and how these affect the conclusions that can be drawn in relation to the original review question | Not Applicable |  |

*From:* Campbell, M., McKenzie, J. E., Sowden, A., Katikireddi, S. V., Brennan, S. E., Ellis, S., ... & Thomson, H. (2020). Synthesis without meta-analysis (SWiM) in systematic reviews: reporting guideline. *BMJ*, *368*. <http://dx.doi.org/10.1136/bmj.l6890>

**Table SM4.** List of Excluded Studies and Reason for Exclusion

| **No.** | **Article** | **Reason for exclusion** |
| --- | --- | --- |
| 1 | Delavar, F., Pashaeypoor, S., & Negarandeh, R. (2020). The effects of self-management education tailored to health literacy on medication adherence and blood pressure control among elderly people with primary hypertension: A randomized controlled trial. Patient education and counseling, 103(2), 336-342. | 2 |
| 2 | Campbell, Z. C., Dawson, J. K., Kirkendall, S. M., McCaffery, K. J., Jansen, J., Campbell, K. L., ... & Webster, A. C. (2022). Interventions for improving health literacy in people with chronic kidney disease. Cochrane Database of Systematic Reviews, (12). | 5 |
| 3 | Lee, S. J., Song, M., & Im, E. O. (2017). Effect of a Health Literacy–Considered Diabetes Self-Management Program for Older Adults in South Korea. Research in Gerontological Nursing, 10(5), 215-225. | 1 |
| 4 | Blancafort Alias, S., Monteserín Nadal, R., Moral, I., Roqué Fígols, M., Rojano i Luque, X., & Coll-Planas, L. (2021). Promoting social capital, self-management and health literacy in older adults through a group-based intervention delivered in low-income urban areas: results of the randomized trial AEQUALIS. BMC Public Health, 21, 1-12. | 4 |
| 5 | Van den Broucke, S., Van der Zanden, G., Chang, P., Doyle, G., Levin, D., Pelikan, J., ... & Riemenschneider, H. (2014). Enhancing the effectiveness of diabetes self-management education: the diabetes literacy project. Hormone and Metabolic Research, 46(13), 933-938. | 2 |
| 6 | Sudhakar, S., Aebi, M. E., Burant, C. J., Wilson, B., Wenk, J., Briggs, F. B., ... & Sajatovic, M. (2020). Health literacy and education level correlates of participation and outcome in a remotely delivered epilepsy self-management program. Epilepsy & Behavior, 107, 107026. | 2 |
| 7 | Hossein Mirzaee Beni, Z., Maasoumi, R., Pashaeypoor, S., & Haghani, S. (2022). The effects of self-care education based on the health literacy index on self-care and quality of life among menopausal women: a randomized clinical trial. BMC Women's Health, 22(1), 452. | 2 |
| 8 | Park, J., Kim, S. H., & Kim, J. G. (2020). Effects of message framing and health literacy on intention to perform diabetes self-care: a randomized controlled trial. Diabetes research and clinical practice, 161, 108043. | 2 |
| 9 | Coll-Planas, L., Blancafort, S., Rojano, X., Roqué, M., & Monteserín, R. (2018). Promoting self-management, health literacy and social capital to reduce health inequalities in older adults living in urban disadvantaged areas: protocol of the randomised controlled trial AEQUALIS. BMC Public Health, 18, 1-10. | 5 |
| 10 | Tsaousi, F., Bouloukaki, I., Christodoulakis, A., Ierodiakonou, D., Tzanakis, N., & Tsiligianni, I. (2024). A Chronic Obstructive Pulmonary Disease Self-Management Intervention for Improving Patient-Reported Outcomes in Primary Care in Greece. Medicina, 60(3), 377. | 2 |
| 11 | Wu, Y., Wen, J., Wang, X., Wang, Q., Wang, W., Wang, X., ... & Cong, L. (2023). Chinese Community Home-Based Aging Institution Elders’ Self-Management of Chronic Non-Communicable Diseases and Its Interrelationships with Social Support, E-Health Literacy, and Self Efficacy: A Serial Multiple Mediation Model. Patient preference and adherence, 1311-1321. | 2 |
| 12 | Boonasa, K., Panpanit, L., Banharak, S., Kumniyom, N., & Chanaboon, S. (2022). Literacy Enhancement Program among Older People Receiving Hemodialysis: Feasibility and Acceptability. Pacific Rim International Journal of Nursing Research, 26(3), 474-487. | 4 |
| 13 | Taheri, E., Araban, M., Ghanbari, S., & Moradi Kalboland, M. (2022). The Relationship between Health Literacy and Rate of Receiving Integrated and Comprehensive Geriatric Care Program with Self-care Ability in Elderly Women. Journal of Health Literacy, 7(1), 45-55. | 5 |
| 14 | Lopez-Olivo, M. A., Foreman, J. T., Leung, C., Lin, H. Y., Westrich-Robertson, T., Hofstetter, C., ... & Suarez-Almazor, M. E. (2022). A randomized controlled trial evaluating the effects of social networking on chronic disease management in rheumatoid arthritis. In Seminars in arthritis and rheumatism (Vol. 56, p. 152072). WB Saunders. | 2 |
| 15 | Rubin, D. J., Gogineni, P., Deak, A., Vaz, C., Watts, S., Recco, D., ... & Allen, S. (2022). The Diabetes Transition of Hospital Care (DiaTOHC) pilot study: a randomized controlled trial of an intervention designed to reduce readmission risk of adults with diabetes. Journal of Clinical Medicine, 11(6), 1471. | 2 |
| 16 | Kamath, D. Y., Abdullakutty, J., Granger, B. B., Kulkarni, S., Bhuvana, K. B., Salazar, L. J., ... & Xavier, D. (2023). A randomized controlled trial evaluating a theory driven, complex intervention centered on task sharing and mobile health to improve selfcare and outcomes in heart failure–The PANACEA-HF RCT: Design and rationale. American Heart Journal Plus: Cardiology Research and Practice, 34, 100310. | 2 |
| 17 | Cornely, R. M., Subramanya, V., Owen, A., McGee, R. E., & Kulshreshtha, A. (2022). A mixed-methods approach to understanding the perspectives, experiences, and attitudes of a culturally tailored cognitive behavioral therapy/motivational interviewing intervention for African American patients with type 2 diabetes: a randomized parallel design pilot study. Pilot and Feasibility Studies, 8(1), 107. | 2 |
| 18 | Bas-Sarmiento, P., Fernández-Gutiérrez, M., Poza-Méndez, M., Marín-Paz, A. J., Paloma-Castro, O., Romero-Sánchez, J. M., & Team, A. (2022). Development and effectiveness of a mobile health intervention in improving health literacy and self-management of patients with multimorbidity and heart failure: protocol for a randomized controlled trial. JMIR Research Protocols, 11(4), e35945. | 5 |
| 19 | Bailey, S. C., Pack, A. P., Wismer, G., Calderon, N., Velazquez, E., Batio, S., ... & Grobman, W. A. (2023). Promoting REproductive Planning And REadiness in Diabetes (PREPARED) Study protocol: a clinic-randomised controlled trial testing a technology-based strategy to promote preconception care for women with type 2 diabetes. BMJ open, 13(11), e078282. | 2 |
| 20 | Amoozadeh, B., Parandeh, A., Khamseh, F., & Goharrizi, M. A. S. B. (2023). The effect of culturally appropriate self-care intervention on health literacy, health-related quality of life and glycemic control in Iranian patients with type 2 diabetes: A controlled randomized clinical trial. Iranian Journal of Nursing and Midwifery Research, 28(3), 293-299. | 2 |
| 21 | Lopez‐Olivo, M. A., Lin, H., Rizvi, T., Barbo Barthel, A., Ingleshwar, A., des Bordes, J. K., ... & Suarez‐Almazor, M. E. (2021). Randomized controlled trial of patient education tools for patients with rheumatoid arthritis. Arthritis care & research, 73(10), 1470-1478. | 2 |
| 22 | Valerio, M. A., Peterson, E. L., Wittich, A. R., & Joseph, C. L. (2016). Examining health literacy among urban African-American adolescents with asthma. Journal of Asthma, 53(10), 1041-1047. | 2 |
| 23 | Kerber, A., Beintner, I., Burchert, S., & Knaevelsrud, C. (2021). Does app-based unguided self-management improve mental health literacy, patient empowerment and access to care for people with mental health impairments? Study protocol for a randomised controlled trial. BMJ open, 11(7), e049688. | 2 |
| 24 | Konerding, U., Redaèlli, M., Ackermann, K., Altin, S., Appelbaum, S., Biallas, B., ... & Stock, S. (2021). A pragmatic randomised controlled trial referring to a Personalised Self-management SUPport Programme (P-SUP) for persons enrolled in a disease management programme for type 2 diabetes mellitus and/or for coronary heart disease. Trials, 22, 1-17. | 2 |
| 25 | Nokes, K. M., & Reyes, D. M. (2019). Do brief educational sessions increase electronic health literacy of low-income persons living with HIV/AIDS?. CIN: Computers, Informatics, Nursing, 37(6), 315-320. | 2 |
| 26 | Nelson, L. A., Spieker, A., Greevy, R., LeStourgeon, L. M., Wallston, K. A., & Mayberry, L. S. (2020). User engagement among diverse adults in a 12-month text message–delivered diabetes support intervention: results from a randomized controlled trial. JMIR mHealth and uHealth, 8(7), e17534. | 2 |
| 27 | Lopez-Olivo, M. A., Des Bordes, J. K. A., Lin, H., Rizvi, T., Volk, R. J., & Suarez-Almazor, M. E. (2020). Comparison of multimedia and printed patient education tools for patients with osteoporosis: a 6-month randomized controlled trial. Osteoporosis International, 31, 857-866. | 2 |
| 28 | Harris, K., Newby, C., Mosler, G., Steed, L., Griffiths, C., & Grigg, J. (2022). School-based self-management intervention using theatre to improve asthma control in adolescents: a pilot cluster-randomised controlled trial. Pilot and Feasibility Studies, 8(1), 67. | 2 |
| 29 | Wattanawong, O., Prachaiboon, T., Meererksom, T., Rattanapitoon, N. K., Rattanapitoon, S. K., Banchonhattakit, P., ... & Kophachon, T. (2021). OVCCA web application as supplementary material to facilitate health literacy regarding carcinogenic human liver fluke: a randomized controlled trial in Thailand. Asian Pacific Journal of Cancer Prevention: APJCP, 22(9), 3045. | 4 |
| 30 | Salisbury, C., O'Cathain, A., Edwards, L., Thomas, C., Gaunt, D., Hollinghurst, S., ... & Montgomery, A. A. (2016). Effectiveness of an integrated telehealth service for patients with depression: a pragmatic randomised controlled trial of a complex intervention. The Lancet Psychiatry, 3(6), 515-525. | 2 |
| 31 | Hickman, R. L., Clochesy, J. M., & Alaamri, M. (2021). Effects of an eHealth intervention on patient-provider interaction and functional health literacy in adults with hypertension. SAGE Open Nursing, 7, 23779608211005863. | 4 |
| 32 | Khanal, M. K., Bhandari, P., Dhungana, R. R., Bhandari, P., Rawal, L. B., Gurung, Y., ... & Courten, B. D. (2021). Effectiveness of community-based health education and home support program to reduce blood pressure among patients with uncontrolled hypertension in Nepal: A cluster-randomized trial. PloS one, 16(10), e0258406. | 2 |
| 33 | Kim, K. B., Han, H. R., Huh, B., Nguyen, T., Lee, H., & Kim, M. T. (2014). The effect of a community-based self-help multimodal behavioral intervention in Korean American seniors with high blood pressure. American journal of hypertension, 27(9), 1199-1208. | 2 |
| 34 | Hung, J. Y., Chen, P. F., Livneh, H., Chen, Y. Y., Guo, H. R., & Tsai, T. Y. (2017). Long-term effectiveness of the Diabetes Conversation Map Program: A prepost education intervention study among type 2 diabetic patients in Taiwan. Medicine, 96(36), e7912. | 2 |
| 35 | Wonggom, P., Du, H., & Clark, R. A. (2018). Evaluation of the effectiveness of an interactive avatar‐based education application for improving heart failure patients’ knowledge and self‐care behaviours: a pragmatic randomized controlled trial protocol. Journal of advanced nursing, 74(11), 2667-2676. | 2 |
| 36 | Lynch, E. B., Liebman, R., Ventrelle, J., Keim, K., Appelhans, B. M., Avery, E. F., ... & Fogelfeld, L. (2014). Design of the Lifestyle Improvement through Food and Exercise (LIFE) study: a randomized controlled trial of self-management of type 2 diabetes among African American patients from safety net health centers. Contemporary clinical trials, 39(2), 246-255. | 2 |
| 37 | Pardhan, S., Upadhyaya, T., Smith, L., Sharma, T., Tuladhar, S., Adhikari, B., ... & Sapkota, R. (2023). Individual patient-centered target-driven intervention to improve clinical outcomes of diabetes, health literacy, and self-care practices in Nepal: A randomized controlled trial. Frontiers in Endocrinology, 14, 1076253. | 2 |
| 38 | Creber, R. M., Patey, M., Lee, C. S., Kuan, A., Jurgens, C., & Riegel, B. (2016). Motivational interviewing to improve self-care for patients with chronic heart failure: MITI-HF randomized controlled trial. Patient education and counseling, 99(2), 256-264. | 2 |
| 39 | Laybourne, A. H., Morgan, M., Watkins, S. H., Lawton, R., Ridsdale, L., & Goldstein, L. H. (2015). Self-management for people with poorly controlled epilepsy: Participants' views of the UK Self-Management in epILEpsy (SMILE) program. Epilepsy & Behavior, 52, 159-164. | 2 |
| 40 | Van der Hout, A., Holtmaat, K., Jansen, F., Lissenberg-Witte, B. I., van Uden-Kraan, C. F., Nieuwenhuijzen, G. A. P., ... & Verdonck-de Leeuw, I. M. (2021). The eHealth self-management application ‘Oncokompas’ that supports cancer survivors to improve health-related quality of life and reduce symptoms: which groups benefit most? Acta Oncologica, 60(4), 403-411. | 2 |
| 41 | Jafree, S. R., Muzammil, A., Burhan, S. K., Bukhari, N., & Fischer, F. (2023). Impact of a digital health literacy intervention and risk predictors for multimorbidity among poor women of reproductive years: Results of a randomized-controlled trial. Digital Health, 9, 20552076221144506. | 4 |
| 42 | Song, Y., Beltran Puerta, J., Medina-Aedo, M., Canelo-Aybar, C., Valli, C., Ballester, M., ... & Heijmans, M. (2023, December). Self-Management Interventions for Adults Living with Type II Diabetes to Improve Patient-Important Outcomes: An Evidence Map. In Healthcare (Vol. 11, No. 24, p. 3156). MDPI. | 2 |
| 43 | Tan, E., Khoo, J., Gani, L. U., Malakar, R. D., Tay, T. L., Tirukonda, P. S., ... & Tang, T. Y. (2019). Effect of multidisciplinary intensive targeted care in improving diabetes mellitus outcomes: a randomized controlled pilot study–the integrated diabetes education, awareness and lifestyle modification in Singapore (IDEALS) program. Trials, 20, 1-10. | 2 |
| 44 | Schaffler, Jamie, et al. "The effectiveness of self-management interventions for individuals with low health literacy and/or low income: a descriptive systematic review." Journal of general internal medicine 33 (2018): 510-523. | 2 |
| 45 | Fransen, Mirjam P., Christian von Wagner, and Marie-Louise Essink-Bot. "Diabetes self-management in patients with low health literacy: ordering findings from literature in a health literacy framework." Patient education and counseling 88.1 (2012): 44-53. | 2 |
| 46 | Wang, Chenli, et al. "The effect of health literacy and self-management efficacy on the health-related quality of life of hypertensive patients in a western rural area of China: a cross-sectional study." International journal for equity in health 16 (2017): 1-11. | 2 |

*Legend.* *Exclusion/inclusion criteria in PICO format.*

| **PICO** | **Inclusion criteria** | **Exclusion criteria** |
| --- | --- | --- |
| 1. *Participants*   **[P]** | Patients diagnosed with a chronic disease* > 18 years | Children, adolescents or exclusively older adults, with a chronic disease |
| 1. *Interventions*   **[I]** | Health literacy interventions delivered in patients, aiming to strengthen, support or increase self-management | Interventions not declared to be a health literacy intervention |
| 1. *Comparisons*   **[C]** | No treatment control groups, attention control groups (participants receive some other attention) or standard care control groups, post-test. | Studies without a comparison |
| 1. *Outcomes*   **[O]** | Self-management or one of the domains of self-management | Did not consider at least one of the SM domains in their outcomes |
| 1. Design | Randomized controlled trials (RCTs) and quasi-experimental studies. | Protocols, systematic reviews, cross-sectional designs, or qualitative research |
| 1. Language | English and Spanish | All other languages |

*(*) Chronic diseases were defined according to criteria from the World Health Organization (WHO) and the Centers for Disease Control and Prevention (CDC). The diseases included but were not limited to type 2 diabetes; cardiovascular diseases; chronic respiratory diseases; musculoskeletal disorders; chronic kidney disease; chronic liver disease; and cancer.*

| **Table SM5.** Characterization of interventions based on health literacy aimed at SM of chronic diseases | | | | | | | | |  |  |
| --- | --- | --- | --- | --- | --- | --- | --- | --- | --- | --- |
| [ID] | Source | Country  design | Chronic disease  Participants  Age | Intervention^†^ | Setting  Duration  Follow-up | Related variables | Limitations | Suggestions or  future directions | Power Analysis | Risk of bias |
| [1] | Ağralı & Akyar (2022) | Turkey  RCT | **T2DM**  Age not reported  (*M* = 56.9)  CG: 60  IG: 60  (*n* = 120) | HL intervention comprised six weekly 60-minute face-to-face sessions in group, followed by six weeks of counselling. The intervention utilized interactive videos, images and an educational booklet. The content included disease information, medication, and lifestyle. Cultural responsiveness (-) | Primary care  12 weeks  12 weeks | Perceived susceptibility, severity, barriers, benefits | (1) HL scale did not have any cut-off value for low/high HL | (1) Patients’ HL levels should be considered to increase knowledge and self-efficacy and reduce perceived barriers | A priori, unclear  sample size  n = 94/  Met | Low |
| [2] | Borge et al. (2024) | Norway  RCT | **Chronic obstructive pulmonary disease**  ≥18 years  CG: 63  IG: 64  (*n* = 127) | HL-informed intervention comprised weekly in-person home visits for 8 weeks, along with monthly phone counselling follow-ups. The intervention involved open-ended questions and discussions. The content covered lifestyle, medication, and treatment. Cultural responsiveness (-) | Home-based care  8 weeks  6 months and one year | Health services costs, QOL,  e-health literacy, stress,  perceived helpfulness | (1) Unblinded data sampling  (2) Language barrier  (3) Missing data | (1) HL intervention can be applicable to other chronic conditions | A priori 0.8  sample size  n = 60 in each group/  Met | Low |
| [3] | Han et al. (2019) | USA  single-arm pre- and post-test design | **T2DM**  ≥18 years  IG: 19  (*n* = 19) | PLAN 4 Success-Diabetes included four weekly 1-hour in-person group sessions, two home visits, and monthly phone counselling for 24 weeks follow-ups. The intervention utilized multimedia resources, role-playing and practical exercises. The content included diabetes HL, treatment, lifestyle, and well-being. Cultural responsiveness (+) | Hospital setting  4 weeks  Baseline, 12 weeks, and 24 weeks | Social support, depression quality of life | (1) Small sample size  (2) No control group  (3) Limited generalizability  (4) Non-validated instrument | (1) Incorporate social determinants into interventions for low-income patients | Not adequately powered | Moderate |
| [4] | Barkhordari-Sharifabad et al. (2021) | Iran  clinical trial | **Heart failure**  35-70 years  CG: 28  IG: 28  (*n* = 56) | iSpring Play App. Each week for one month, a topic link was provided. The intervention involved photos, animation, and short videos. The content covered lifestyle, problem-solving, and well-being. Cultural responsiveness (-) | Hospital setting (e-learning)  4 weeks  No follow-up | No reported | (1) Short period to investigate the effect of HL on self-care | (1) To use longer periods of time to confirm the effects of interventions | A priori 0.8,  sample size  n = 28 in each group/  Met | Moderate |
| [5] | Guo et al. (2023) | Taiwan  quasi-experimen | **T2DM**  20-65 years  CG: 36  IG: 96  (*n* = 132) | Mobile eHL education program comprised of two modules: one on using health websites (30-60 minutes) and another on using diabetes Apps with practical exercises. The content were internet basics, navigation, evaluation, and app usage. Cultural responsiveness (-) | Hospital setting (e-learning)  No reported; two modules  3 months | Mobile eHealth literacy,  self-rated health | (1) Self-report  (2) No randomization  (3) Unmeasured confounders  (4) Small sample size | (1) Mobile eHL fits chronic care  (2) To explore health technology use  (3) To explore group and individual education  (4) Larger sample size | N/R | Moderate |
| [6] | Hakimzadeh & Adib-Hajbaghery, M. (2021). | Iran  quasi-experimental | **Ischemic heart diseases**  18-65 years  CG: 25  IG: 25  (*n* = 50) | HL training included two face-to-face sessions in group. The intervention utilized an educational booklet, and a social media group for ongoing education and exchange. The content covered disease information, lifestyle and medication. Cultural responsiveness (-) | Hospital setting  3 weeks  No follow-up | Treatment adherence | (1) Small sample size  (2) Unblinded intervention group  (3) Observer bias (author who implemented the intervention was also the one how gathered the data) | (1) Larger, multicenter and blinded studies | A priori 0.8  sample size  n = 24 in each group/  Met | High |
| [7] | Hung et al. (2017) | Taiwan  quasi-experimental | **T2DM**  39-80 years  CG: 46  IG: 49  (*n* = 95) | Diabetes Conversation Map Program (DCMP), a weekly 1.5-hour in-person group program with phone follow-ups. The intervention utilized interactive videos, images and discussions.  The content covered diabetes HL, diet and exercise, insulin use, risk factors and foot care. Cultural responsiveness (-) | Hospital setting  7 weeks  3 months | Depressive symptoms | (1) Limited generalizability  (2) Quasi-experimental design may weaken internal validity  (3) Unmeasured confounders | (1) DCMP concepts might apply to other to patients with other chronic conditions | A priori 0.8,  sample size  n = 34 in each group/  Met | Moderate |
| [8] | Kim et al. (2015) | Korea  RCT | **T2DM**  ≥35 years  CG: 125  IG: 125  (*n* = 250) | HL intervention comprised weekly 2-hour sessions in person  over 6 weeks, and monthly telephone counselling. The intervention utilized multimedia resources, role-playing, and discussions. The content covered diabetes HL, problem-solving, and self-belief. Cultural responsiveness (+) | Independent non-profit community agency  6 weeks  Baseline, 3, 6, 9, and 12 months | Quality of life | (1) Single-center program targeting only one ethnic minority group  (2) Insufficient data on cost-effectiveness | (1) HL intervention can be applicable to ethnic groups  (2) Proven model for linguistically isolated ethnic communities | A priori 0.8,  sample size  n = 105 in each group/  Met | Moderate |
| [9] | Leong et al. (2022) | Taiwan  RCT | **T2DM**  ≥20 years  IG: 90  CG: 91  (*n* = 181) | Three videos weekly and care messages every two weeks on social media for 3 months. The intervention utilized quizzes and electronic resources. The content covered diabetes basics, daily care, nutrition, and medications. Cultural responsiveness (-) | Hospital setting (e-learning)  3 months  No follow-up | Patients’ attitudes toward diabetes | (1) Limited generalizability  (2) Brief program  (3) Self-report and recall bias  (4) Video watch duration and attentiveness not confirmed  (5) Economic value not assessed | (1) Video helps when in-person education is not possible  (2) To enhance telemedicine in other chronic diseases  (3) Include diverse populations and longer durations | A priori 0.8,  sample size  n = 80 in each group/  Met | Moderate |
| [10] | Moura et al. (2019) | Brazil  Quasi- experimental pre- and post-test design | **T2DM**  30-69 years  IG: 55  (*n* = 55) | Nursing education intervention consisted of three weekly face-to-face meetings of 60 minutes, using visual materials and guided practices. The content covered diabetes knowledge and self-care practices. Cultural  responsiveness (-) | Family Health setting  3 weeks  No follow-up | No reported | (1) Limited generalizability  (2) Low adherence to the intervention  (3) No control group  (4) Single post-intervention revaluation | (1) Educational interventions must address content that meets the HL needs  (2) To include time-series evaluations for longitudinal follow-up | N/R | Moderate |
| [11] | Michou et al. (2022) | Canada  RCT | **Rheumatoid arthritis**  54-71 years  CG: 52  IG: 57  (*n* = 109) | The intervention included a 40-minute DVD on rheumatoid arthritis along with a group online teleconference, using multimedia resources, Q&A forum, posters, and patient testimonial. The content covered disease, treatments, lifestyle, costs, physiotherapy, well-being. Cultural responsiveness (-) | Hospital setting (e-learning)  3 months  Follow-up at 6 months | Belief about Medication, behavioural intention | (1) Non-validated instrument  (2) Scales had insufficient internal consistency | (1) Videos offer accurate SM information  (2) To enable innovative projects like a disease passport | A priori 0.8,  sample size  n = 71 in each group/  Not met | Moderate |
| [12] | Sriklo et al. (2023) | Thailand  RCT | **T2DM**  35-39 years  CG: 32  IG: 32  (*n* = 64) | The program consisted of four weekly 60-minute face-to-face sessions, utilizing multimedia resources and role-playing. The content covered, T2DM communication, media literacy, and decision-making. Cultural responsiveness (-) | Hospital setting  4 week  12 weeks | No reported | (1) SM at 8-12 weeks post-program may not be sufficient to show sustained change  (2) Limited generalizability | (1) Further testing in diverse settings with different samples  (2) Use of innovative technology | A priori 0.8,  sample size  n = 32 in each group/  Met | Low |
| [13] | Whittemore et al. (2020) | Mexico  Randomised wait-list control pilot | **T2DM**  21-70 years  CG: 21  IG: 26  (*n* = 47) | Diabetes self-management program comprised of 7 weekly face-to-face group sessions, 6 months of using daily texts, pictures, and follow-up calls. The content covered self-monitoring, diet, physical activity, medication, and well-being. Cultural responsiveness (+) | Public primary healthcare centre 7 weeks + 6 months of messages  Biweekly follow-up calls | Depressive symptoms | (1) Small sample  (2) Selection bias | (1) Theory-based text messages to support SM over time | N/R | Low |
| [14] | Zeidi et al. (2021) | Iran  RCT | **T2DM**  ≥45 years  CG: 83  IG: 83  (*n* = 166) | The program covered five face-to-face training sessions of 45 minutes, using lectures, role playing, focus group and practical exercises. The content covered disease symptoms, HL, self-care, attitude change, self-efficacy. Cultural responsiveness (-) | Public primary healthcare centre  2 months  No follow-up | Behavioral intention, subjective norms | (1) Lack of follow up  (2) Self-report and recall bias | (1) To include one- and two-year follow-up  (2) To include objective behavioural measures (HbA1c, BMI). | A priori 0.8,  sample size  n = 83 in each group/  Met | Moderate |

**Note**: ^†^ Intervention: mode, delivery strategies and content. Abbreviations: IG, Intervention Group; CG, Control group; SMBG, Self-Monitoring of Blood Glucose; HbA1c, glycated hemoglobin; QOL, Quality of Life; eHL, eHealth literacy;

N/R, no reported; Risk of bias based on JBI Quality Assessment Checklist. Cultural responsiveness refers to the extent to which interventions incorporate cultural, ethnic, and contextual characteristics of the target population in their design, implementation, and evaluation.

# **Table SM6a**. Risk of bias of the selected Quasi-Experimental Studies based on the JBI Checklist

| **First Author, Year** | **Q1** | **Q2** | **Q3** | **Q4** | **Q5** | **Q6** | **Q7** | **Q8** | **Q9** | **Overall Risk of bias** |
| --- | --- | --- | --- | --- | --- | --- | --- | --- | --- | --- |
| Han (2019) | **+** | **+** | **+** | **+** | **+** | **+** | **+/-** | **+** | **-** | Moderate |
| Barkhordari (2021) | **+** | **+** | **+** | **+** | **+** | **+** | **+** | **-** | **+** | Moderate |
| Guo (2023) | **+** | **+** | **-** | **+** | **+** | **+** | **+** | **+** | **-** | Moderate |
| Hakimzadeh (2021) | **+** | **+** | **+** | **+** | **+** | **+** | **+/-** | **-** | **+/-** | High |
| Hung (2017) | **+** | **+** | **+** | **+** | **+** | **+** | **+/-** | **-** | **+** | Moderate |
| Moura (2019) | **+** | **+** | **+** | **+** | **+** | **+** | **+** | **+/-** | **+/-** | Moderate |

|  | |
| --- | --- |
| Q1. Is it clear in the study what is the “cause” and what is the “effect”? |  |
| Q2. Was there a control group? |  |
| Q3. Were participants included in any comparisons similar? |  |
| Q4. Were the participants included in any comparisons receiving similar treatment/care, other than the exposure or intervention of interest? |  |
| Q5. Were there multiple measurements of the outcome, both pre and post the intervention/exposure? |  |
| Q6. Were the outcomes of participants included in any comparisons measured in the same way? |  |
| Q7. Were outcomes measured in a reliable way? |  |
| Q8. Was follow-up complete and if not, were differences between groups in terms of their follow-up adequately analyzed? |  |
| Q9. Was appropriate statistical analysis used? |  |

*From:* Barker, T. H., Habibi, N., Aromataris, E., Stone, J. C., Leonardi-Bee, J., Sears, K., ... & Munn, Z. (2024). The revised JBI critical appraisal tool for the assessment of risk of bias for quasi-experimental studies. *JBI Evidence Synthesis, 22*(3), 378-388. <http://dx.doi.org/10.11124/JBIES-23-00268>

# **Table SM6b.** Risk of bias of the selected RCT based on the JBI Checklist

| **First Author, Year** | **Q1** | **Q2** | **Q3** | **Q4** | **Q5** | **Q6** | **Q7** | **Q8** | **Q9** | **Q10** | **Q11** | **Q12** | **Q13** | **Risk of bias** |
| --- | --- | --- | --- | --- | --- | --- | --- | --- | --- | --- | --- | --- | --- | --- |
| Agrali (2022) | **+** | **+** | **+** | **?** | **+** | **+/-** | **+** | **+** | **+** | **+** | **+** | **+** | **+** | Low |
| Borge (2024) | **+** | **+** | **+** | **-** | **-** | **-** | **+** | **+** | **+** | **+** | **+** | **+** | **+** | Low |
| Kim (2015) | **+** | **?** | **+** | **?** | **?** | **?** | **+** | **+** | **+** | **+** | **+** | **+** | **+** | Moderate |
| Leong (2022) | **+** | **+** | **+/-** | **-** | **-** | **-** | **+** | **+** | **+** | **+** | **+** | **+** | **+** | Moderate |
| Michou (2022) | **+** | **+** | **-** | **-** | **-** | **-** | **+** | **+** | **+** | **+** | **+** | **+** | **+** | Moderate |
| Sriklo (2023) | **+** | **+** | **+** | **-** | **-** | **+** | **+** | **+** | **+** | **+** | **+** | **+** | **+** | Low |
| Whittemore (2020) | **+** | **+** | **+** | **+** | **+** | **+** | **+** | **+** | **+** | **+** | **+** | **+/-** | **+** | Low |
| Zeidi et al. (2021) | **+** | **?** | **+** | **?** | **?** | **?** | **+** | **+** | **+** | **+** | **+** | **+** | **+** | Moderate |

| Q1. Was true randomization used for assignment of participants to treatment groups? |
| --- |
| Q2. Was allocation to treatment groups concealed? |
| 1. Were treatment groups similar at the baseline? |
| 1. Were participants blind to treatment assignment? |
| 1. Were those delivering treatment blind to treatment assignment? |
| 1. Were outcomes assessors blind to treatment assignment? |
| 1. Were treatment groups treated identically other than the intervention of interest? |
| 1. Was follow up complete and if not, were differences between groups in terms of their follow up adequately described and analyzed? |
| 1. Were participants analyzed in the groups to which they were randomized? |
| 1. Were outcomes measured in the same way for treatment groups? |
| 1. Were outcomes measured in a reliable way? |
| 1. Was appropriate statistical analysis used? |
| 1. Was the trial design appropriate, and any deviations from the standard RCT design accounted for in the conduct and analysis of the trial? |

*From:* Barker, T. H., Stone, J. C., Sears, K., Klugar, M., Tufanaru, C., Leonardi-Bee, J., ... & Munn, Z. (2023). The revised JBI critical appraisal tool for the assessment of risk of bias for randomized controlled trials. *JBI evidence synthesis, 21*(3), 494-506. http://dx.doi.org/10.11124/JBIES-22-00430

| **Table SM7.** Theoretical foundations and conceptualization of HL and SM | | | | | | |
| --- | --- | --- | --- | --- | --- | --- |
| [ID] | Author | Theoretical Model | HL conceptualization | | SM conceptualization | |
|  |  |  | HL definition | HL measurement  Components | SM definition | SM measurement  Components |
| 1 | Ağralı & Akyar (2022) | Health Belief Model,  Rosenstock [15] | Cognitive and social skills needed to access, understand, and use health information, such as communication, and critical skills Nutbeam [16] | Health Literacy Scale  Ishikawa et al. [17]  Functional, communicative,  critical | N/P | Diabetes Management Self-Efficacy Scale  Bijl et al. [18]  Medical |
| 2 | Borge et al. (2024) | Motivational interviewing  Miller [19] | Ability to understand, access, use, and remember health information  to promote and maintain health WHO [20] | Health Literacy Questionnaire  Osborne et al. [21]  Functional, communicative,  critical | N/P | Health Education Questionnaire Osborne et al. [22]  Medical |
| 3 | Han et al.  (2019) | von Wagner’s  model  Wagner et al. [23] | Capacity to obtain, process, and understand basic health information  and services to make appropriate health decisions US [24] | Literacy Assessment  in Diabetes  Nath et al. [25]  Functional | N/P | Diabetes self-care index  (Created for the study)  Medical |
| 4 | Barkhordari-Sharifabad et al. (2021) | N/P | N/P | N/P | N/P | Self-care and heart failure index  Riegel et al. [26]  Medical |
| 5 | Guo et al.  (2023) | Framework on  eHL  Schulz et al. [27] | eHL/ capacity to assess health information from electronic sources and utilize the acquired knowledge to resolve health issues  Lin et al. [28] | eHealth Literacy Scale  Norman et al. [29]  Functional | N/P | Diabetes self-care behavior 36-item questionnaire  Parchman et al. [30]  Medical, emotional |
| 6 | Hakimzadeh & Adib-Hajbaghery, (2021) | N/P | To obtain, process, understand and use health information and services needed to make appropriate health related decisions Coyle et al. [31] | N/P | N/P | Self-care behavior questionnaire  Seyed et al. [32]  Medical |
| 7 | Hung et al.  (2017) | N/P | N/P | DM health literacy questionnaire  (By study investigators)  Functional | N/P | SMBG  Medical |
| 8 | Kim et al.  (2015) | Predisposing, Reinforcing, and Enabling Constructs in Environmental Diagnosis Model | N/P | Diabetes Knowledge Test  Fitzgerald et al. [32]  Functional | N/P | Summary of Diabetes  Self-Care Activities  scale Toobert et al. [34]  Medical |
| 9 | Leong et al. (2022) | N/P | N/P | Newest Vital Sign (NVS) tool  Weiss et al. [35]  Functional | N/P | Summary of Diabetes  Self-Care Activities Scale  Toobert et al. [34]  Medical |
| 10 | Moura et al. (2019) | N/P | Cognitive and social skills and ability to access, process and understand information and basic health services necessary to make appropriate health decisions  WHO [36] | Summary of Diabetes  Self-Care Activities scale  Toobert et al. [34]  Functional | N/P | Summary of Diabetes Self-Care Activities Scale  Toobert et al. [34]  Medical |
| 11 | Michou et al. (2022) | Theory of Planned Behavior  Ajzen [37] | N/P | BioSecure questionnaire  Gossec et al. [38]  Functional, communicative | N/P | BioSecure questionnaire -self-care. Gossec et al. [38]  Medical, role, emotional |
| 12 | Sriklo et al.  (2023) | Transformative learning Model Mezirow [39] | Cognitive and social skills needed  to access, understand, and use health information, such as communication, and critical skills Nutbeam [16] | Health Literacy Scale  Ishikawa et al. [17]  Functional, communicative, critical | Care responsibility during illness through medical, role, and emotional management  Lorig et al. [40] | SM Behaviours Scale  Lorig et al. [40]  Medical, role, emotional |
| 13 | Whittemore  et al. (2020) | Health Action Process Approach Schwarzer et al. [41] | N/P | N/P | N/P | Summary of Diabetes Self-Care Activities Questionnaire  Toobert et al. [34]  Global Physical Activity Questionnaire  Herrmann et al. [42]  Medical |
| 14 | Zeidi et al. (2021) | Theory of Planned Behaviour (TPB)  Ajzen [37] | Capacity of individuals to acquire,  process, and understand basic health services and information in order to make appropriate health decisions  Ishikawa et al. [17] | Short Test of Functional Health Literacy in Adults  Thomason et al. [43]  Functional | N/P | Summary of Diabetes Self-Care Activities Questionnaire  Toobert et al. [34]  Medical |

**Note.** Abbreviations: HbA1c: glycated haemoglobin, N/P Not provided, SMBG: Self-Monitoring of Blood Glucose

**References**

1. Ağralı H, Akyar İ. The effect of health literacy-based, health belief-constructed education on glycated hemoglobin (HbA1c) in people with type 2 diabetes: a randomized controlled study. *Prim. Care Diabetes.* 2022;16:173-178.

2. Borge CR, Larsen MH, Osborne RH, et al. Impacts of a health literacy-informed intervention in people with chronic obstructive pulmonary disease (COPD) on hospitalization, health literacy, self-management, quality of life, and health costs – A randomized controlled trial. *Patient Educ Couns.* 2024;123:108220.

3. Han H, Nkimbeng M, Ajomagberin O, et al. Health literacy enhanced intervention for inner-city African Americans with uncontrolled diabetes: a pilot study. *Pilot Feasibility Stud.* 2019;5:99.

4. Barkhordari-Sharifabad M, Saberinejad K, Nasiriani K. The effect of health literacy promotion through virtual education on the self-care behaviors in patients with heart failure: a clinical trial. *J. Health Lit.* 2021;6:51-60.

5. Guo S, Lin J, Hsing H, Lee C, Chuang S. The effect of mobile eHealth education to improve knowledge, skills, self-Care, and mobile eHealth literacies among patients with diabetes: development and evaluation study. *J. Med. Internet Res.* 2023;25:e42497.

6. Hakimzadeh Z, Adib-Hajbaghery M. The effects of a health literacy training intervention on self-care behaviors and treatment adherence in patients with ischemic heart diseases. *Eurasian J Med Oncol.* 2021;5:341-349.

7. Hung JY, Chen PF, Livneh H, Chen YY, Guo HR, Tsai TY. Long-term effectiveness of the diabetes conversation map program: a prepost education intervention study among type 2 diabetic patients in Taiwan. *Medicine.* 2018;96:e7912.

8. Kim MT, Kim KB, Huh B, et al. The effect of a community-based self-help intervention: Korean americans with type 2 diabetes. *Am. J. Prev. Med.* 2015;49:726-737.

9. Leong CM, Lee TI, Chien YM, Kuo LN, Kuo YF, Chen HY. Social Media-Delivered Patient Education to Enhance Self-management and Attitudes of Patients with Type 2 Diabetes During the COVID-19 Pandemic: Randomized Controlled Trial. *J. Med. Internet Res.* 2022;24:e31449.

10. Moura N, Lopes B, Teixeira JJ, Oriá M, Vieira N, Guedes M. Literacy in health and self-care in people with type 2 diabetes mellitus. *Rev Bras Enferm.* 2019;72:700-706.

11. Michou L, Julien A, Witteman H, et al. Measuring the impact of an educational intervention in rheumatoid arthritis: an open-label, randomized trial. *Arch. Rheumatol.* 2021;37:169–179.

12. Sriklo M, Tamdee D, Aungwattana S, Kaewthummanukul T. Effects of enhance health literacy through transformative learning program on self-management and hemoglobin A1C level among adults with uncontrolled type 2 diabetes: a randomized controlled trial. *Pac. Rim Int. J. Nurs. Res.* 2023;27:317-333.

13. Whittemore R, Vilar-Compte M, De La Cerda S, et al. Yo puedo vivir sano con diabetes! a self-management randomized controlled pilot trial for low-income adults with type 2 diabetes in mexico city. *Curr. Dev. Nutr.* 2020;4:074.

14. Zeidi IM, Morshedi H, Alizadeh Otaghvar H. A theory of planned behavior-enhanced intervention to promote health literacy and self-care behaviors of type 2 diabetic patients. *J Prev Med Hyg*. 2021;4:601-613

15. Rosenstock IM. The health belief model and preventive health behavior. *Health Educ. Monogr.* 1974;2:354-386.

16. Nutbeam D. Health literacy as a public health goal: a challenge for contemporary health education and communication strategies into the 21st century. *Health Promot. Int.* 2000;15:259-267.

17. Ishikawa H, Takeuchi T, Yano E. Measuring functional, communicative, and critical health literacy among diabetic patients. *Diabetes Car*e. 2008;31:874-879.

18. Bijl J, Poelgeest-Eeltink A, Shortridge-Baggett L. The psychometric properties of the diabetes management self-efficacy scale for patients with type 2 diabetes. *J Adv Nurs.* 1999;30:352-359.

19. Miller WR. Motivational interviewing with problem drinkers. *Behavioural Psychotherapy.* 1983;11:147-172.

20. World Health Organization*. Health Promotion Glossary of Terms 2021*. Geneva; 2021.

21. Osborne RH, Batterham RW, Elsworth GR, Hawkins M, Buchbinder R. The grounded psychometric development and initial validation of the Health Literacy Questionnaire (HLQ). *BMC Public Health*. 2013;13:658.

22. Osborne RH, Elsworth GR, Whitfield K. The health education impact questionnaire (heiQ): an outcomes and evaluation measure for patient education and self-management interventions for people with chronic conditions. *Patient Educ Couns*. 2007;66:192-201.

23. von Wagner C, Steptoe A, Wolf MS, Wardle J. Health literacy and health actions: a review and a framework from health psychology. *Health Educ Behav.* 2008;36:860-877.

24. U.S. Department of Health and Human Services. *Healthy People 2010: Understanding and Improving Health*. Washington, DC: U.S. Department of Health and Human Services; 2000.

25. Nath CR, Sylvester ST, Yasek V, Gunel E. Development and validation of a literacy assessment tool for persons with diabetes. *Diabetes Educ.* 2001;27:857-864.

26. Riegel B, Carlson B, Moser DK, Sebern M, Hicks FD, Roland V. Psychometric testing of the self-care of heart failure index. *J Card Fail.* 2004;10:350–360.

27. Schulz P, Nakamoto K. Patient behavior and the benefits of artificial intelligence: the perils of "dangerous" literacy and illusory patient empowerment. *Patient Educ Couns.* 2013;92:223-228.

28. Lin T, Bautista JR. Understanding the relationships between mHealth apps’ characteristics, trialability, and mHealth literacy. *J. Health Commun.* 2017;22:346-354.

29. Norman CD, Skinner HA. eHealth literacy: essential skills for consumer health in a networked world. *J. Med. Internet Res.* 2006;8:e9.

30. Parchman M, Pugh J, Wang C, Romero R. Glucose control, Self-care behaviors, and the presence of the chronic care model in primary care clinics. *Diabetes Care.* 2007;30:2849-2854.

31. Coyle MK. Depressive symptoms after a myocardial infarction and self-care. *Arch Psychiatr Nurs*. 2012;26:127–34.

32. Seyed Fatemi N, Rafii F, Hajizadeh E, Modanloo M. Psychometric properties of the adherence questionnaire in patients with chronic disease: A mix method study. Koomesh 2018;20:179–91.

33. Fitzgerald JT, Funnell M, Hess G, et al. The reliability and validity of a brief diabetes knowledge test. *Diabetes Care.* 1998;21:706-710.

34. Toobert D, Hampson S, Glasgow R. The Summary of diabetes self-care activities measure: results from 7 studies and a revised scale. *Diabetes Care.* 2000;23:943-950.

35. Weiss BD, Mays MZ, Martz W, et al. Quick assessment of literacy in primary care: the newest vital sign. *Ann. Fam. Med.* 2005;3:514-522.

36. World Health Organization. *Health Promotion Glossary*. Geneva, Switzerland: World Health Organization; 1998.

37. Ajzen I. The theory of planned behavior. *Organ. Behav. Hum. Decis. Process.* 1991;50:179-211.

38. Gossec L, Fautrel B, Flipon É, et al. Safety of biologics: elaboration and validation of a questionnaire assessing patients' self-care safety skills: the bioSecure questionnaire: an initiative of the french rheumatology society therapeutic education section. *Joint Bone Spin*e. 2013;80:471–476.

39. Mezirow J. *Transformative Dimensions of Adult Learning*. San Francisco, Jossey-Bass; 1991.

40. Lorig KR, Holman HR. Self-management education: history, definition, outcomes, and mechanisms. *Ann. Behav. Med.* 2003;*26:*1-7.

41. Schwarzer R, Lippke S, Ziegelmann J. Health action process approach: a research agenda at the freie universität berlin to examine and promote health behavior change. *Zeitschr. Gesundheitspsychol.* 2008;16:157–160.

42. Herrmann SD, Heumann KJ, Der Ananian CA, Ainsworth BE. Validity and reliability of the Global Physical Activity Questionnaire (GPAQ). *Meas Phys Educ Exerc Sci.* 2013;17:221–35.

43. Thomason TR, Mayo AM. A Critique of the Short Test of Functional Health Literacy in Adults. *Clin Nurse Spec.* 2015;29:308–312.
